# The CULTEX RFS: A Comprehensive Technical Approach for the *In Vitro* Exposure of Airway Epithelial Cells to the Particulate Matter at the Air-Liquid Interface

**DOI:** 10.1155/2013/734137

**Published:** 2013-02-07

**Authors:** Michaela Aufderheide, Beat Halter, Niklas Möhle, Dieter Hochrainer

**Affiliations:** ^1^Cultex Laboratories GmbH, Feodor-Lynen-Straße 21, 30625 Hannover, Germany; ^2^Halter Engineering GmbH, Huebstraße 16, 9100 Herisau, Switzerland; ^3^Von der Hardt 16, 57392 Oberkirchen, Germany

## Abstract

The EU Regulation on Registration, Evaluation, Authorization and Restriction of Chemicals (REACH) demands the implementation of alternative methods for analyzing the hazardous effects of chemicals including particulate formulations. In the field of inhalation toxicology, a variety of *in vitro* models have been developed for such studies. To simulate the *in vivo* situation, an adequate exposure device is necessary for the direct exposure of cultivated lung cells at the air-liquid interface (ALI). The CULTEX RFS fulfills these requirements and has been optimized for the exposure of cells to atomized suspensions, gases, and volatile compounds as well as micro- and nanosized particles. This study provides information on the construction and functional aspects of the exposure device. By using the Computational Fluid Dynamics (CFD) analysis, the technical design was optimized to realize a stable, reproducible, and homogeneous deposition of particles. The efficiency of the exposure procedure is demonstrated by exposing A549 cells dose dependently to lactose monohydrate, copper(II) sulfate, copper(II) oxide, and micro- and nanoparticles. All copper compounds induced cytotoxic effects, most pronounced for soluble copper(II) sulfate. Micro- and nanosized copper(II) oxide also showed a dose-dependent decrease in the cell viability, whereby the nanosized particles decreased the metabolic activity of the cells more severely.

## 1. Introduction

Provoked by public pressure and triggered by an increasing number of lethal lung diseases over the last few decades [1, 2], more and more studies in the field of inhalation toxicology now concentrate on the understanding of particle-lung interactions. Investigations of the toxicological effects of inhalable substances on the respiratory tract mainly focus on results from animal experiments based on the OECD guideline 403 on acute inhalation toxicology. So far, only a few *in vitro* alternatives to animal inhalation tests for toxicology have been described [3, 4]. However, none of them is validated or officially accepted by the authorities.

Recent changes in the EU chemical policy, namely, the new Registration, Evaluation, Authorization and Restriction of Chemicals directive (REACH; EC no. 1907/2006), and complaints about the immense number of animals needed to fulfill the requirements of REACH [6] demand the development and implementation of novel *in vitro* technologies—also in the field of inhalation toxicology. In order to evaluate the effects of relevant particulate substances, only classic methodological approaches are available using either suspended or dissolved particles under submerged conditions in cell culture experiments [4, 7]. The main concerns about these test methods are (1) the unrealistic behavior of suspended particles and (2) culture and exposure conditions which do not reflect the situation in the lung. The fact of losing nanosized particles by agglomeration or the uncontrollable behavior of nanosized particles in suspensions may lead to uncertainties in the results [8].

Another major point of the discussion is the transferability of data from animal experiments to the human organism due to species-specific reactions and the generation of false positive or negative results [9].

One of the “first” approaches for direct cell exposure came from Tarkington et al. [10] who conducted the atmosphere via a vertical stream directly over the cultivated test organisms. The system is also based on a biphasic cell culture exposed at an air-liquid interface [11]. The basic idea is to mimic the inhalation cycle *in vitro* by taking into consideration the most important biological and technical aspects. The selection of an appropriate cell model and its cultivation at the air-liquid interface are the basic prerequisites for such a system. On the other hand, the technical implementation should guarantee the direct contact of the test substances with the cells, as well as the homogeneous exposure of the entire cell layer without interfering with the cell viability.

These theoretical concepts led to the development of the CULTEX exposure module in 1999 by Aufderheide and Mohr [12] for the exposure of cultivated cells at the air-liquid interface. The test aerosol is conducted directly over the cells through specially designed inlet nozzles. This setup guarantees a close contact between the test aerosol and the cells without any interference of the cell culture media. The first CULTEX exposure devices were used for the exposure to complex mixtures like cigarette smoke and gases, or in a modified version to analyze the mutagenic potency of airborne materials in the AMES assay [13].

Nowadays, a large number of other air-liquid interface exposure systems are available, ranging from the exposure of two-cell culture plates (6-well), like the ALICE exposure device [14] or a flow-through system [15] to a radial multiwell module [16]. All of these modules have certain advantages and disadvantages and may therefore only be used for a limited test assembly.

The results obtained from exposure studies with the CULTEX RFS module have shown that the interactions between cells and particles are closely linked to the physical and chemical properties of these compounds and have advanced the redesign of the handmade CULTEX glass modules.

The CULTEX Radial Flow System (RFS) presented here overcomes the limitations of its predecessor model and includes all features that are required to realize the exposure of cultivated cells to airborne particles under realistic conditions.

## 2. Material and Methods

### 2.1. Technical Description

**Figure 1:**
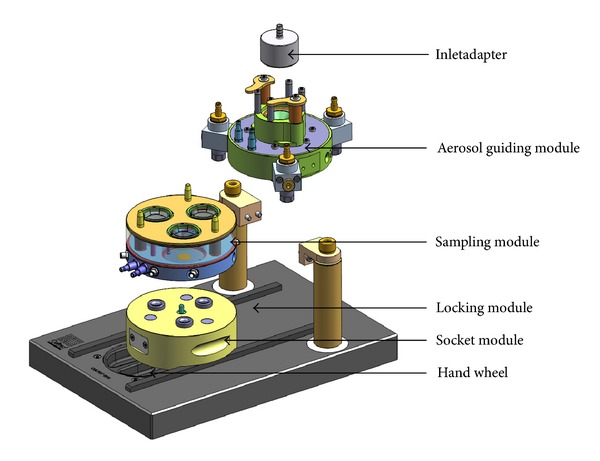
Overview image of the modular CULTEX RFS exposure system. The device is composed of the following components: (1) inlet adapter, (2) aerosol guiding module, (3) sampling module and socket module, and (4) locking module with a hand wheel.

The CULTEX RFS module was designed for exposing adherent growing cells at the air-liquid interface and is a precision instrument, characterized by a modular construction (Figure 1).

**Figure 2:**
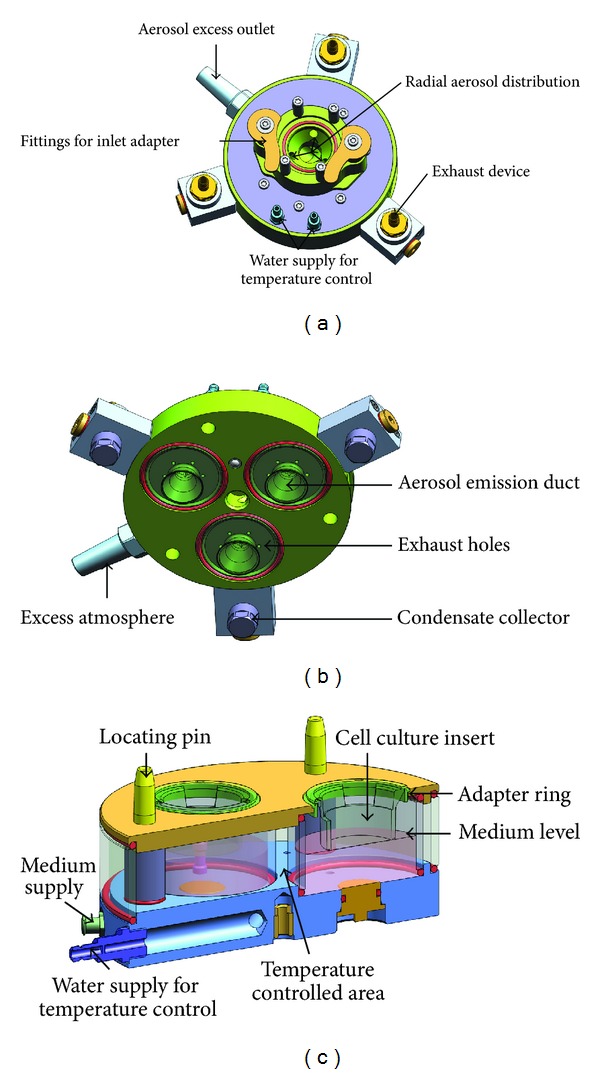
Aerosol guiding module of the CULTEX RFS: (a) top view, (b) bottom view, and (c) sampling module.

**Figure 3:**
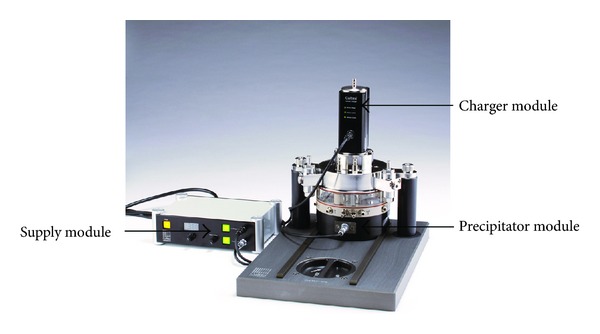
The CULTEX RFS exposure device extended with an electrical deposition device (Cultex EDD) for the increased particle deposition efficiency.

The basic CULTEX RFS consists of the following parts: (1) the inlet adapter which connects the aerosol generation and aerosol guiding module (Figure 1), (2) the aerosol guiding module to conduct and distribute the particles to be deposited on the cell culture inserts in the sampling module (Figures 2(a) and 2(b)), and (3) the sampling and socket module with three exposure chambers where the cell culture inserts or Petri dishes are located (Figures 1 and 2(c)). 

The aerosol guiding module as well as the sampling module can be heated to the appropriate temperature (e.g., 37°C) by the connection to an external water bath. The socket module (4) guides the sampling module on the slide rails of the locking device (5) and serves as a spacer for integrating additional functions, like a control unit for electrostatic precipitation.

In addition to the above-mentioned modular components, the system can also be equipped with special adapters to enable the use of commercial inserts from different suppliers and of different sizes or for Petri dishes. The aerosol emission ducts are adapted correspondingly.

To increase the particle deposition efficiency, notably for nanosized particles, the electrical deposition device (Cultex EDD) can further be integrated into the technical setup (Figure 3).

### 2.2. Test Materials

Dry powder atmospheres were prepared from the substances listed in Tables 1 and 2 and used for the exposure studies.

The substances were pressed into powder cakes by the CULTEX HyP-Hydraulic Press (Cultex Laboratories GmbH, Germany), which allows electronically controlled compression of a high variety of powders by a pneumo-hydraulic cylinder.

### 2.3. Aerosol Generation

**Figure 4:**
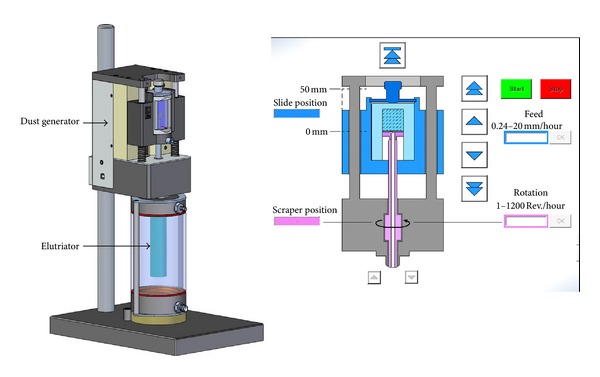
The CULTEX DG (Dust Generator) (Cultex Laboratories GmbH, Germany) enables the generation of aerosols from a powder cake according to Wright [5].

The aerosol was generated from the prepared powder cakes with the CULTEX DG (Dust Generator) (Cultex Laboratories GmbH) according to Wright [5] (Figure 4). The fully computerized generator is able to provide uniform airborne concentrations of dust over a long period of time. 

The highly compressed substances are scraped off by a rotating scraper under standardized controlled conditions (feed rate 0.24 to 20 mm/h, rotation 1 to 800 revs/h). The total exposure time is varied from 15, 30, to 60 minutes to generate different particle concentrations on the cell culture membranes without changing the aerosol generation or any other physical or chemical parameter.

The generated particles were transported to the integrated elutriator by a constant air stream (8 L/min). The elutriator retains undesired large particles (> approx. 8 *μ*m) and serves as a reservoir for a uniform aerosol which is finally drawn through the CULTEX RFS.

**Figure 5:**
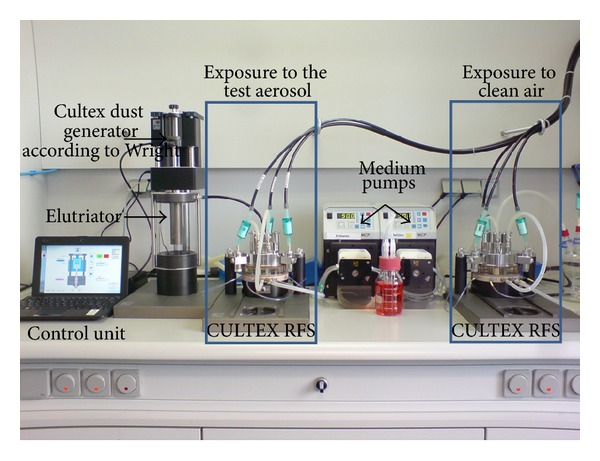
Experimental setup for exposing cultivated cells at the air-liquid interface to particles. The components of the exposure station are the particle generator according to Wright, the elutriator, two CULTEX RFS devices for exposure to particles and clean air and medium pumps for the automatic nutrient supply.

The complete experimental setup is shown in Figure 5 consisting of the particle generation unit, two CULTEX RFS devices, and two pumps for the medium supply. The cells are exposed to the test aerosol and clean air (process control) in parallel, in order to preclude process-related reactions which might interfere with the substance-specific effects.

### 2.4. CFD Analysis

The aerosol flow within the experimental setup was simulated and optimized by means of Computational Fluid Dynamics (CFD) software (ANSYS CFX, ANSYS Incorporated).

### 2.5. Particle Number and Mass Distribution

The determination of the particle number and mass distribution was conducted with an *Aerodynamic Particle Sizer* APS (3321/TSI Incorporated) in the size range of 0.5 to 20 *μ*m. By accelerating particles through a nozzle and optical time-of-flight measurement of the particles, they are classified into 51 logarithmic size ranges between 0.523 and 20 *μ*m. 

### 2.6. Particle Deposition

The deposition of the particles was analyzed by gravimetric methods, using the precision balance (SE2-F filter ultra-microbalance, Sartorius).

**Figure 6:**
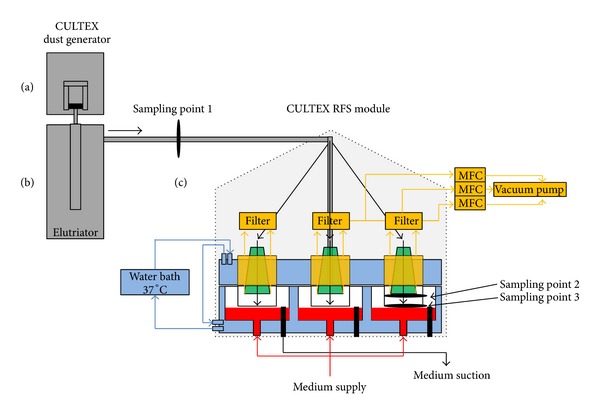
Schematic overview of the CULTEX system for exposing cultivated cells to particles at the air-liquid interface. Components of the exposure station: (a) particle generator according to Wright (CULTEX Dust Generator); (b) elutriator: glass tube with vertical upward flow, where large particles are removed from the aerosol due to sedimentation; (c) CULTEX RFS modules for exposure to particles and synthetic air. The sampling points (1–3) for the test aerosols in the overall stream are marked.

In preliminary tests, the particle mass concentration within the exposure system was analyzed at three sampling points (sampling point 1: 200 seconds, sampling point 2 + 3: 60 minutes) to determine appropriate exposure times for the corresponding test particles (Figure 6) and to check for uniform particle distribution. The first sampling point was located directly after the elutriator, the second at the outlet port of the aerosol emission duct to estimate the particle mass entering the exposure chamber, and the third at the insert membrane surface to measure the particle mass deposited on the cultivated cells. The particle mass was determined by collecting the particles on filter pads (glass fiber filter/GF-A/Macherey-Nagel), which were weighed before and after the exposure over a constant period (15 minutes). This approach allows the analysis of the deposition efficiency of each test atmosphere within the exposure module. Under these experimental conditions, the deposited particle numbers on the cell cultures can only be calculated by taking into consideration the measurements of the *Aerodynamic Particle Sizer* APS (3321/TSI Incorporated) and the system-specific deposition capacities (Aufderheide et al. [17], Supplementary Material).

### 2.7. Cell Cultivation and Exposure

For particle exposure experiments, the human lung adenocarcinoma epithelial cell line A549 (ATCC number: CCL-185) was used [18, 19]. The cells were grown in Dulbecco's MEM (Biochrom FG 0145, Germany) supplemented with 10% fetal calf serum and gentamicin (5 *μ*g/mL). 

A549 cells were seeded onto microporous membranes (growth area: 4.2 cm²) of cell culture inserts (0.4 *μ*m pore size, BD Biosciences) with a density of 1∗10^5^ cells/cm^2^ and cultivated submerged in a cell culture medium. After 24 hours, the apical medium was removed from the confluent cell layers and the direct exposure at the air-liquid interface with the CULTEX RFS was started. The cells were exposed either to the test substances (deposition rate: 25 *μ*g/cm²/15 min) or clean air (process control) for 15, 30, and 60 minutes. The incubator control cultures were cultivated at the air-liquid interface in the incubator during the exposure period.

### 2.8. WST Assay

The aim of our study was the characterization of the exposure device, limited to the functional description of the system. Accordingly, we used only one biological endpoint, the metabolic activity of the cells, to demonstrate dose-dependent cytotoxic reactions, not investigating further into the mechanisms behind these effects. 

After a postincubation time of 24 hours (air-liquid interface; 37°C/5% CO_2_), the particle-exposed cells and the control cultures (incubator control: unexposed cells, process control: clean air-exposed cells) were analyzed for cell viability by using the WST-1 assay for the mitochondrial activity according to the manufacturer's instructions (Roche Diagnostics, Germany). The data of the exposed cells were normalized to the values of the clean air control. The incubator controls were not considered, because they did not show significant differences to the clean air controls.

### 2.9. Statistical Analysis

The results of three independent tests with three samples each is expressed as mean ± SD. A Student's *t*-test was performed to analyze whether the differences between the mean values of the three exposure times are significant [20].

## 3. Results

### 3.1. Deposition Efficiency

The CULTEX RFS was designed for exposing adherent cells at the air-liquid interface to airborne materials like gases, complex mixtures, and particles. When dealing with particulate matter especially, questions arise concerning the deposition efficiency of such an exposure device.

The basic and theoretical considerations forming the basis of the efficiency of the system are already described by Aufderheide et al. ([17], Electronic Supplementary Material).

### 3.2. CFD Analysis—Flow Conditions within the System

**Figure 7:**
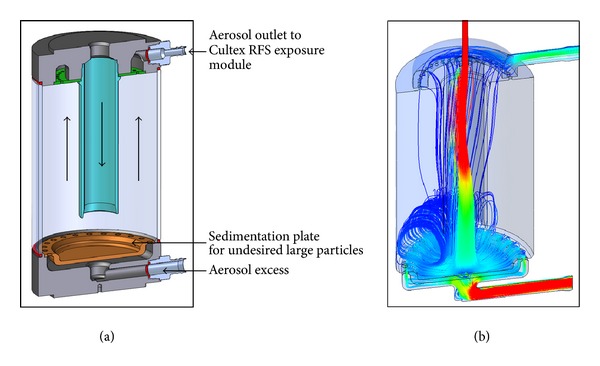
Sectional view of the elutriator (a) and streamline plot calculated by CFD Analysis (b).

**Figure 8:**
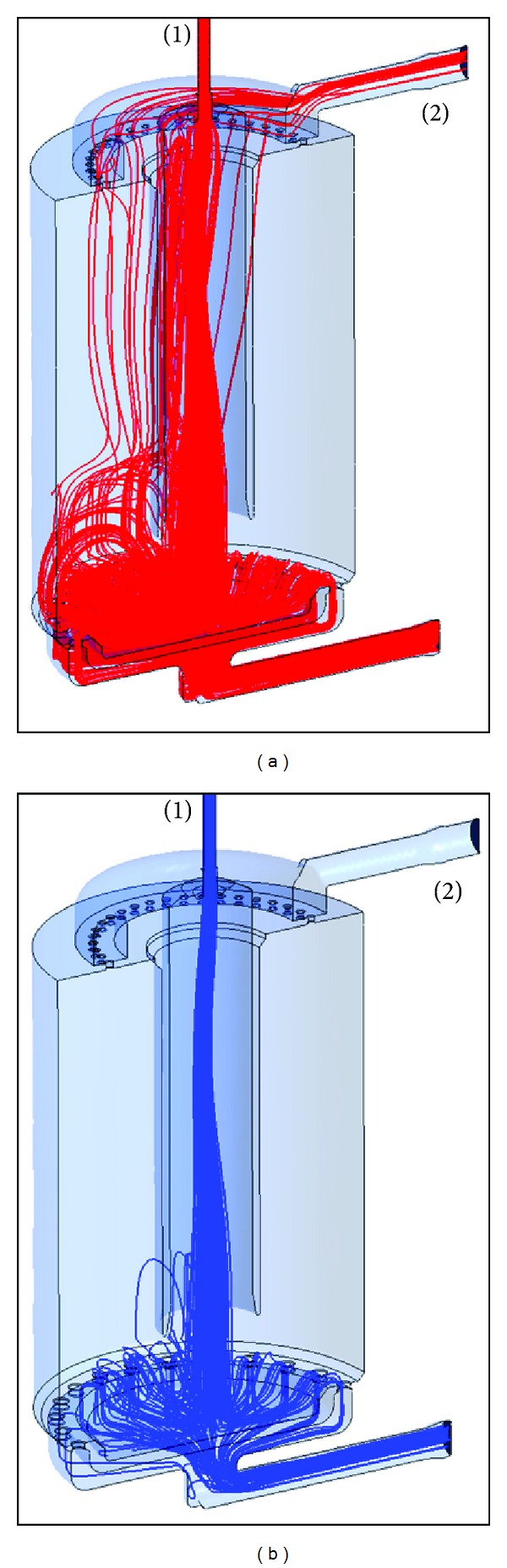
Particle track simulation for 2 *μ*m (a) and 10 *μ*m (b) particles. 8 L/min inlet flow rate (1) and 1.09 L/min outlet low rate (2).

**Figure 9:**
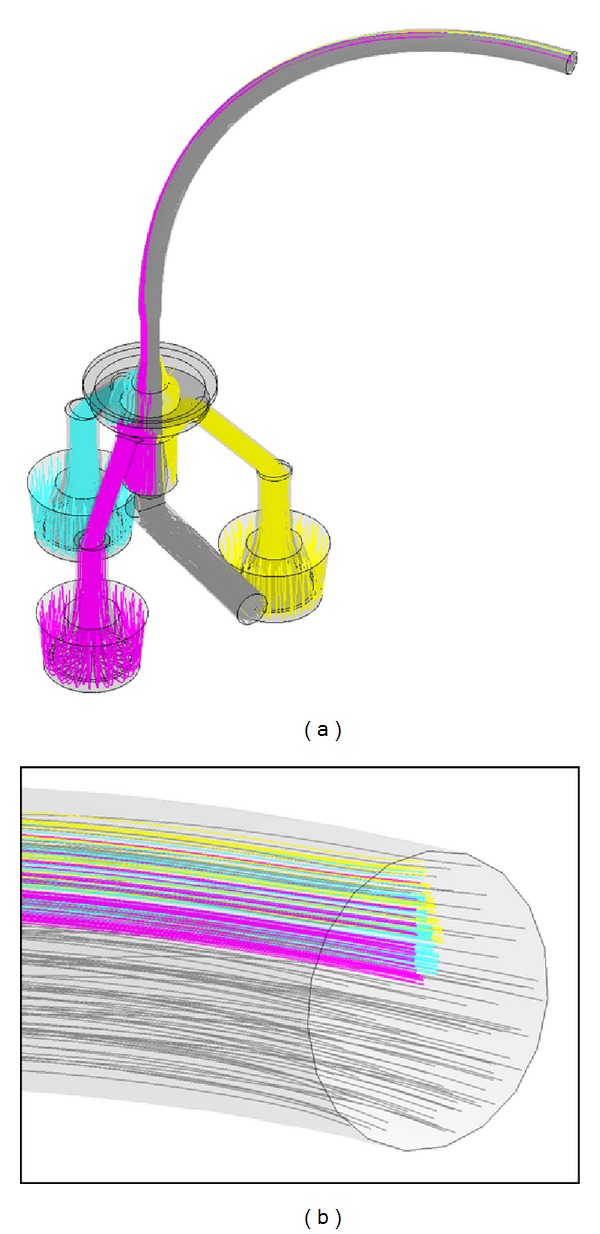
(a) Backtracking of flow lines from deposition chambers and excess outlet to the beginning of the aerosol feeding tube. (b) Flow lines running to the deposition chambers start at specific locations at the beginning of the feeding tube.

When developing the CULTEX RFS and its peripheral devices, a key to achieve uniform particle deposition on the cell cultures was the simulation and optimization of the particle flow within the system by means of CFD analysis (Figures 7 to 9).

Figure 7 shows a cross-section through the elutriator (a) and a streamline velocity plot (b) of the CFD analysis. The basic principle of an elutriator consists of separating small (light) from large (heavy) particles in a vertically upward directed stream. The elutriator features an additional outlet at the bottom for discharging excess aerosol, as aerosol generation may require higher flow rates (e.g., 8 L/min) than those for the CULTEX RFS exposure module (e.g., 1.6 L/min). The streamline plot shows a curl in the lower zone of the device but a uniform upstream above, which is essential for a reliable particle separation. The results are based on flow rates of 8.0 L/min at the inlet, 1.09 L/min at the outlet, and 6.91 L/min at the aerosol excess outlet. Further calculations with the same flow rate at the inlet but 1.59 L/min at the aerosol outlet showed no substantial differences.

Figure 8 shows particle trajectory simulations with particle sizes of 2 *μ*m (a) and 10 *μ*m (b). While small particles are transported upwards to the aerosol outlet and the Cultex RFS module, large particles remain in the elutriator. The major portion of the particles is carried to the excess outlet, as a flow rate of only 1.09 L/min from totally 8 L/min was conducted to the module in this simulation.

When testing prototypes of the Cultex RFS module, the distribution patterns between the three deposition chambers and within the individual chambers showed considerable differences. CFD calculations of the gas flow lines and particle trajectories resulted in the following essential findings.

Figure 9(a) shows the aerosol flow channels within the Cultex RFS module including a curved aerosol feeding tube with 6 mm diameter and 200 mm length. The flow lines represent an aerosol flow of 1590 mL/min in the feeding tube, which is divided into three minor flows of 30 mL/min leading to the deposition chambers and an excess flow of 1500 mL/min. Backtracking a defined number of particles from the deposition chamber to the beginning of the curved aerosol feeding tube (which is equal to the elutriator outlet) showed that these particles originate from specific locations in the tube profile (Figure 9(b)). Further calculations showed that the specific locations are sensitive to changes in tube bending radius, tube length, or flow rate. As the particle concentration and particle size distribution at the beginning of the feeding tube is usually nonuniform across the tube profile, the distribution pattern in the deposition chambers are consequently also nonuniform.

**Figure 10:**
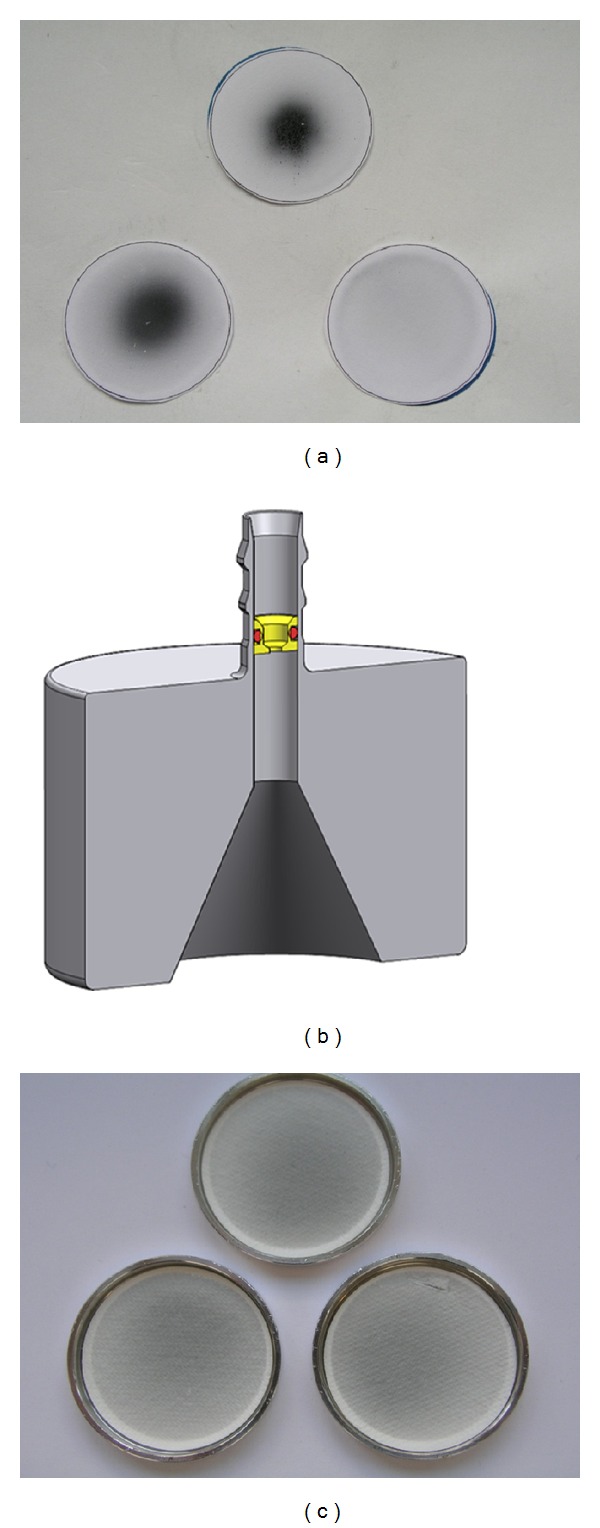
(a) Unequal deposition of copper(II) oxide microparticles on filter membranes after a 60** **min exposure. (b) Inlet-adapter with an integrated jet nozzle to avoid unequal particle deposition within the CULTEX RFS module. (c) Uniform deposition of copper(II) oxide on filter membranes after a 60** **min exposure with a jet nozzle.

An optimal solution to completely avoid these undesired effects was the integration of a jet nozzle into the inlet adapter of the Cultex RFS module.  Figure 10(a) shows the deposition of copper(II) oxide micro on insert membranes without using a jet nozzle. The integration of a jet nozzle into the inlet adapter (Figure 10(b)) resulted in a homogenous distribution of the particles on the insert membranes (Figure 10(c), Table 3).

### 3.3. Dose-Response Relationship

After optimizing the deposition characteristics within the CULTEX RFS module, A549 cells were exposed to lactose monohydrate (process control), copper(II) sulfate (soluble substance), copper(II) oxide micro as well as copper(II) oxide nanoparticles (insoluble) for 15, 30, and 60 minutes. The particle generation was adjusted for each substance to result in a particle deposition of 25 *μ*g/cm² (low dose) during an exposure time of 15 minutes. The concentration (low effect level, LOEL) is based on an interdisciplinary European project in which the cytotoxic potency of a variety of particles was analyzed with different cell types under submersed culture conditions [21]. The conditions for the particle generation had to be adjusted in preliminary experiments due to substance-specific variations. 24 hours after exposure, the cell viability was measured. The values obtained for the particle-exposed cultures were normalized to the clean air-exposed cells.

The results are shown in Figures 11, 12, 13, 14, 15, 16, 17, and 18. Generally the clean air-exposed cells (process control) showed no significant reduction in the cell viability in comparison with the incubator control. Accordingly, the exposure process itself had no influence on the metabolic activity of the cells.

The exposure of the cell cultures to the test compounds showed, dependent on the chemical and physical properties of the particulate atmosphere, considerable differences in the cytotoxic response among the three exposure times. The comparison of the 15, 30, and 60 minutes exposures in a Student's *t*-test demonstrates significant differences with a 5% error probability. No significant differences could be obtained for lactose monohydrate (30 minutes compared to 15 minutes) and copper(II) oxide micro-sized (60 minutes compared to 30 minutes) due to the low number of tests.

The exposure of A549 cells to lactose monohydrate (negative substance) led to a slight decrease in the cell viability of the cells after 15 minutes (94% of the clean air control). By increasing the exposure time to 60 minutes, the metabolic activity of the cells was reduced by 30% in comparison to the clean air control. At that point, the cultures were covered by a dense layer of the particulate matter thus pointing to an overload effect.

**Figure 12:**
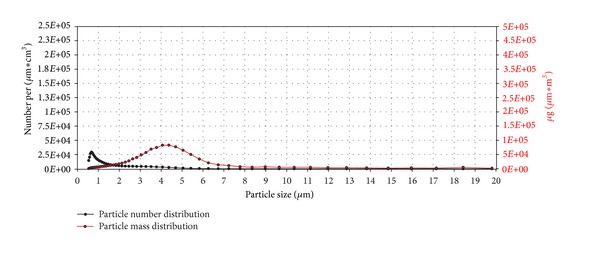
Particle number (black line) and particle mass distribution (red line) of the generated lactose monohydrate aerosol entering the exposure module. The analysis was performed with an Aerodynamic Particle Sizer (TSI Inc.).

The particle number (black line) and mass distribution (red line) of the lactose monohydrate aerosol dependent on particle size are shown in Figure 12. The units number, respectively, mass per *μ*m∗cm^3^ may be unfamiliar at first glance. As the curves are based on particle counts, classified to 51 particle size intervals, the counts have to be divided not only through the volume but also through the interval width to get the required values for particle distribution curves.

The particle number distribution curve exhibits its peak value at a particle size of 0.7 *μ*m while the particle *mass* distribution curve shows its peak value at a particle size of 4.2 *μ*m due to the greater mass of larger particles.

The exposure of the cell populations to copper(II) sulfate led to a pronounced decrease in the cell viability after 15 minutes 19% of the clean air control. By increasing the time to 60 minutes, the metabolic activity was reduced by more than 91% compared to the clean air control. 

**Figure 14:**
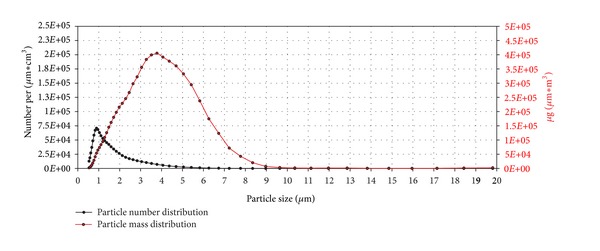
Particle number (black line) and particle mass distribution (red line) of the generated copper(II) sulfate aerosol entering the exposure module. The analysis was performed with an Aerodynamic Particle Sizer (TSI Inc.).

The peak for the particle number distribution was at 0.9 *μ*m and the peak for the particle mass distribution was at 3.8 *μ*m (Figure 14). Both particle number and particle mass were about five times higher than for lactose monohydrate.

**Figure 15:**
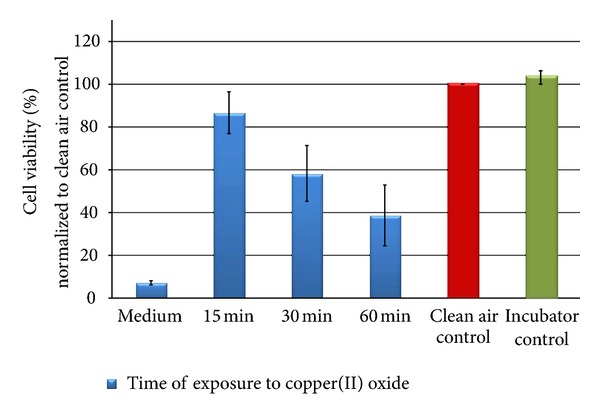
Relative cell viability of A549 cells after exposure to copper(II) oxide microparticles normalized to the clean air control dependent on time (15, 30, and 60 min).

The results after the exposure of A549 cells to copper(II) oxide micro also indicated a dose-dependent decrease of the metabolic activity over the exposure time (Figure 15). The decrease of cell viability after 60 minutes exposure (61% reduction in comparison to the synthetic air control) is significantly higher than for lactose monohydrate but not as clear as for copper(II) sulfate.

**Figure 16:**
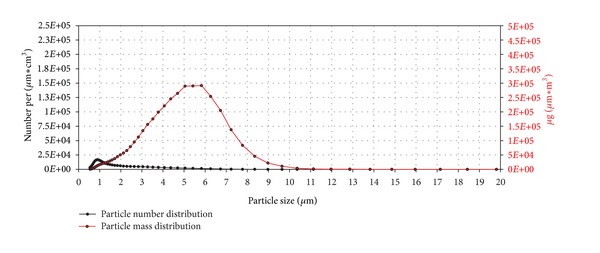
Particle number (black line) and particle mass distribution (red line) of the generated copper(II) oxide microaerosol entering the exposure module. The analysis was performed with an Aerodynamic Particle Sizer (TSI Inc.).

In comparison with copper(II) sulfate, the particle number distribution of copper(II) oxide micro also showed a peak at 0.9 *μ*m, but with a more than four times lower number of particles (Figure 16). The values for the particle mass distribution were about 25% lower compared to copper(II) sulfate, indicating that the substances mostly differ in their content of small particles.

The cell viability of the A549 cells was significantly reduced after the exposure to copper(II) oxide nanoparticles. After 15 minutes, the metabolic activity was reduced to less than 50% compared to the clean air and incubator control. A particle mass of 100 *μ*g/cm^2^ (60 minutes) led to a reduction in the metabolic activity of 85%, indicating a higher cytotoxic effect by the nanopowder compared to the micro.

Copper(II) oxide nano does not differ strongly from copper(II) oxide micro in numbers of sub-*μ*m particles. The nanopowder exhibits much lower mass distribution values, however, due to few particles in the size range over 1 *μ*m. 

## 4. Discussion

Due to changes in the EU regulations concerning the approval of chemical substances (Regulation (EU) no. 1907/2006) and the ongoing demand for alternative methods, new cell systems and exposure techniques were developed and characterized, also in the field of inhalation toxicology. The latter should meet special requirements with regard to the cell type and the type of exposure. 

The biological test systems include bronchial (Calu-3, 16HBE14o-, BEAS-2B) and alveolar epithelial (A549) cell lines, mostly from tumors as well as human primary cells isolated from different regions of the respiratory tract [22–25]. At the moment primary cell cultures are mostly studied under mechanistic aspects (differentiation, cellular interactions) [26] and are not used routinely for screening methods to address acute toxicity. Therefore, the studies are mostly performed with cell lines like the alveolar epithelial cell line A549, which allows the generation of stable cultures (undifferentiated). The advantage of these cultures is the delivery of stable, reproducible, and significant data for the calculation of dose-response curves as well as the definition of key values (effective dose: EC_50_).

The exposure of cultivated cells from the respiratory tract for studying the effects of airborne substances represents a challenge with regard to the experimental design. The exposure of cells under conventional submerged conditions, mostly with soluble test substances, shows a variety of shortcomings like the interference of the test atmosphere with medium components, unrealistic exposure conditions including uncertain effective doses for gases and particles. Therefore, several approaches have been made for the development of exposure systems (Table 4) at the air-liquid interface [3, 12, 14, 17, 27–35]. Under such conditions, the cells are in direct contact with the test aerosol, which is conducted through the exposure device to the cells. The exposure systems have to fulfill cell-specific requirements, meaning the maintenance of the cell cultures during the exposure process by medium supply and the establishment of a cell-specific environment (pH value, 37°C). 

All described systems have taken into consideration those basic requirements. The inserts are located in medium-containing chambers, connected with a medium supply for intermittent or continuous medium exchange. In the system described by Adamson et al. [36], 4 culture vessels are placed together in a chamber filled up with medium to the bottom of the cell culture inserts. In the CULTEX RFS, and also the CULTEX glass modules [27, 28], the inserts are housed separately and are supplied individually with nutrients, as independent exposure chambers within one module. The medium level is adjusted via an overflow tube to establish comparable conditions in all three chambers.

The basic principle of cellular exposure to airborne materials is based on the treatment of the cultivated cells at the air-liquid interface (ALI). The experimental setup to realize a direct contact between the cells and the test atmosphere differs considerably in the different systems, which are listed in Table 4. A limited number of devices favor the exposure of the cultures to an aerosol passing through a box or exposure chamber [35, 36], but most of the exposure systems prefer a stream directed towards the cell culture to realize a close contact between the test atmosphere and the cell surface for depositing particles. Gaseous compounds can be studied in all systems due to the homogeneous distribution of their test atmosphere, whereas the exposure of particulate materials is based mostly on a directed aerosol flow. A comparative study of the different test systems is limited due to the limited availability of the modules and the absence of information.

The CULTEX glass modules as well as the RFS are designed to establish an incoming flow which is directed immediately via emission ducts to the surface of the cells to guarantee a close contact with the test atmosphere, both qualitatively and quantitatively. In comparison with the glass modules, the aerosol guiding module of the RFS has been optimized concerning the uniform distribution of the incoming test atmosphere to the three exposure chambers, thus stabilizing the whole exposure process. In the glass modules, the aerosol is guided linearly above the module and the sampling points for the test atmosphere are arranged in succession. In the case of gaseous compounds, the homogeneous distribution of the atmosphere is not limited, but airborne particles belong to another category, especially with regard to their aerosol physical properties. A linear flow path above the glass module leads to a concentration gradient, which may result, due to the sequentially arranged sampling points, at different exposure levels. In contrast, the CULTEX RFS module is characterized by a central inlet for the test atmosphere into the exposure device, wherefrom the aerosol is distributed into the chambers and the particles deposited on the cell cultures. The resulting data are characterized by a small standard deviation within a test or for multiple experiments. Another relevant advantage of the new system is the adjustment of the medium level, which ensures a comparable microenvironment for all cultures. The level in the Cultex RFS is controlled by special overflow tubes to stabilize the sensitive microclimate around the cells. An autonomous medium supply for each cell culture insert offers the opportunity to test different medium additives without interaction between the three test chambers. The new modular design of the Cultex RFS guarantees a high flexibility in working with different types of cell culture inserts or even Petri dishes (for the AMES assay) by using special adapters.

The efficiency of the exposure process depends on the deposition efficiency and represents, especially in the case of fine and ultrafine particles (nano particles), one of the main challenges [17]. The deposition efficiency of the particles in most air-liquid interface systems is based on sedimentation and diffusion. Accordingly, a characterization of the test aerosol with regard to the particle number and particle mass dependent on the particle size is one of the main requirements to judge the biological activity of the airborne material. Due to changes within the aerosol during the generation process and due to particle-particle interactions, the primary particle size should only be used as a basic indication. In this context, particle loss within the system and agglomeration of the particles has to be taken into account. 

In the literature, the deposition efficiency rate of *in vitro *exposure devices is described inconsistently. Theoretical considerations and experimental exposure data with ultrafine carbonaceous model particles with a CMD of 95 nm resulted in an efficiency of 2% [37]. 

The combination of such air-liquid interface exposure systems with an electrical charger and precipitator could improve particle deposition. Experimental data from Savi et al. [16] showed that the deposition efficiency can be increased to 30% with this type of setup without causing a cytotoxic effect on the exposed cells. First results obtained with the Cultex RFS in combination with an electrical deposition device (Cultex EDD) indicated that the efficiency for particles which are not deposited by sedimentation or diffusion can be increased up to 95% (data not shown).

The outstanding importance of particle size and mass for the deposition efficiency highlights the importance of a controlled and stable generation of particulate atmospheres as well as the behavior of such particles in an exposure device.

To obtain more insights into the flow conditions within our Cultex RFS module, CFD analysis was conducted by taking into consideration all components of the experimental setup. CFD simulations included the particle distribution in the tubing system (connection between the elutriator and the exposure module) and the exposure module itself. Here, we found that inhomogeneous particle distributions propagate over long distances due to laminar flow conditions. The integration of a jet nozzle into the inlet adapter enabled a homogeneous particle distribution and deposition on the insert membranes as shown in Figure 10 at the example of copper(II) oxide micro. The CFD analysis provides a good method to simulate the trajectories of the particles from generation to deposition. The consideration of different experimental conditions like the air flow rate or dimensions of the connecting tubes allows the selection of the appropriate experimental design to enhance the efficiency of the exposure system.

Besides the experimental setup, the chemical and physical properties of the particles highly influence their deposition efficiency. To compare the biological activity of the different copper compounds, the particle mass concentration per area was adjusted for each substance to establish a comparable particle mass deposition (deposition rate: 25 *μ*g/cm²/15 min). A549 cells were exposed for 15, 30, and 60 minutes at the air-liquid interface to the different test atmospheres and the metabolic activity of the cells was analyzed as an estimate for cytotoxicity. In comparison to the incubator control, the cells that were exposed to clean air showed no reduction in the cell viability, indicating that the exposure procedure itself had *no effect* on this analyzed endpoint. As a negative substance control we used lactose, which induced no considerable cell damage (13% after an exposure time of 30 minutes). At the end of the exposure period, the cultures were covered by a dense particle layer and a further reduction in cell viability (37% of the clean air control) was measured probably due to an overload effect.

In comparison with lactose monohydrate, all copper compounds induced significant dose-related effects. The cytotoxic signal correlated strongly with the chemical and physical properties of the test compound. Copper(II) sulfate, as soluble compound, induced a pronounced cytotoxic effect already after an exposure time of 15 minutes (reduction of cell viability of 80%). Comparable effects could also be observed for the insoluble copper(II) oxide compounds, but to a lesser extent. As described by Karlsson and coworkers [38], the exposure of the cells to both substances resulted in a dose-dependent decrease in the cell viability by increasing the particle concentration, whereby the copper(II) oxide nanomaterial appeared to be more harmful to the cells than copper(II) oxide micro, based on the exposed particle mass. In the literature, nanoparticles are repeatedly described to be more potent in causing a cellular damage than microparticles [38–40], but there is also evidence that there is no difference in their biological activity [41–43]. In agreement with the above-mentioned studies, our direct exposure studies with nanosized copper(II) oxide exhibited a stronger cytotoxicity than the micro-sized particles. Most of the studies on micro- or nanosized particles have been conducted under submersed conditions. Ahamed and coworkers also used A549 cells, which were treated with CuO nanoparticles (NP) suspended in medium (0, 10, 25, and 50 *μ*g/mL for 24 hours) [44]. CuO NPs significantly decreased cell viability in a dose-dependent manner. The highest concentration (50 *μ*g/mL) induced a reduction in viability of 52%, whereas the directly exposed A549 cells showed a decrease of more than 75%, pointing to a more efficient contact and interaction between deposited particles and the lung cells. Investigations of Karlsson et al. support this assessment [38, 45].

Concerning the number of particles per cm^2^ for each substance, A549 cells were exposed in the case of copper(II) oxide nano to more particles in comparison to copper(II) oxide micro. Accordingly, the higher cytotoxicity may also be a result of the higher number of smaller particles coming into contact with the surface of the cells. 

In summary, our results show that an efficient and stable cell exposure system like the Cultex RFS module allows a reproducible analysis of dose-dependent reactions to airborne materials. The cell cultures can be exposed to the generated particulate atmospheres, characterized with regard to particle size and mass distribution, under controlled conditions thus favoring the generation of valid data for the calculation of key values (effective dose). These data can be compared with animal or clinical data and offer the possibility to verify the relevance and meaningfulness of these *in vitro* studies.

## Figures and Tables

**Figure 11:**
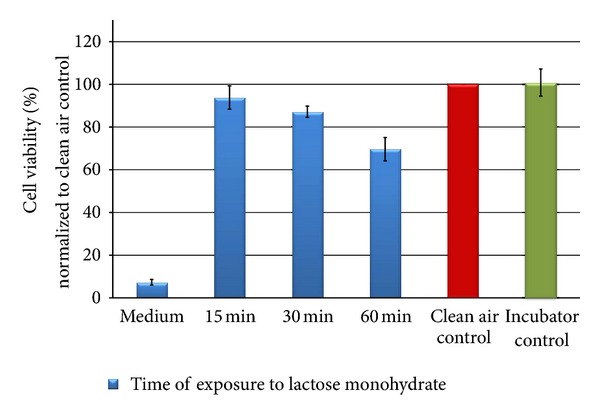
Relative cell viability of A549 cells after exposure to lactose monohydrate particles normalized to the clean air control dependent on time (15, 30, and 60** **min).

**Figure 13:**
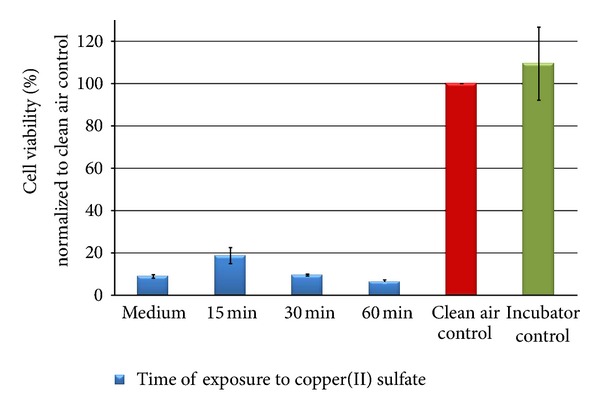
Relative cell viability of A549 cells after exposure to copper(II) sulfate particles normalized to the clean air control dependent on time (15, 30, and 60** **min).

**Figure 17:**
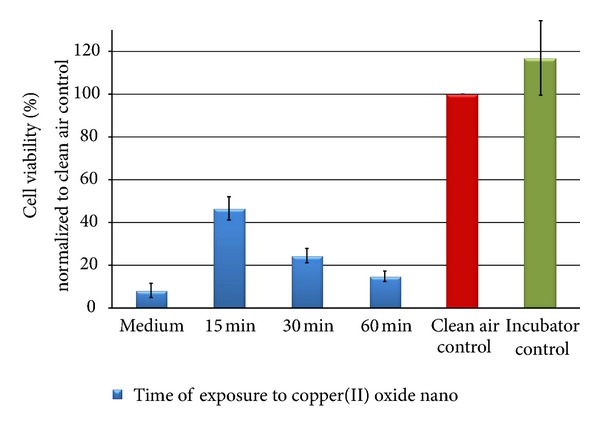
Relative cell viability of A549 cells after exposure to copper(II) oxide nanoparticles normalized to the clean air control dependent on time (15, 30, and 60 min).

**Figure 18:**
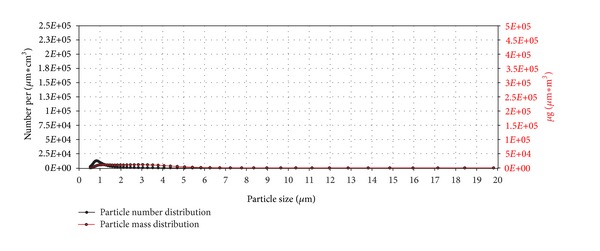
Particle number (black line) and particle mass distribution (red line) of the generated copper(II) oxide nano aerosol entering the exposure module. The analysis was performed with an Aerodynamic Particle Sizer (TSI Inc.).

**Table 1:** Substances used for the generation of dry powder atmospheres.

Substance	Producer/catalogue number	Primary particle size
Lactose monohydrat	Fluka/61341	Not available

Copper(II) oxide nano	Ionic Liquids Technologies GmbH/NO-0031-HP	40–80 nm

Copper(II) oxide mikro	Sigma Aldrich/20844-1	5 *μ*m

Copper(II) sulfat	Sigma Aldrich/12852	Not available

**Table 2:** Conditions for the generation of powder cakes and particulate atmospheres.

Substance	Powder cake generation	Particle generation	
	Pressure (bar)	Scraper (rev/h)	Feed rate (mm/h)
Lactose monohydrat	110	800	10.0
Copper(II) oxide nano	82	800	2.5
Copper(II) oxide micro	82	800	2.0
Copper(II) sulfate	82	800	4.5

**Table 3:** Deposition of copper(II) oxide microparticles on filter membranes after an exposure time of 60 minutes with a jet nozzle in the inlet adapter.

60 minutes exposure of copper(II) oxide	Chamber 1	Chamber 2	Chamber 3
Weight gain of filter paper (*μ*g)	530	529	549
Weight gain of filter paper (*μ*g)	529	537	521
Weight gain of filter paper (*μ*g)	548	553	548

**Table 4:** Exposure systems for exposing cultivated cells at the air-liquid interface.

Exposure system	Electrostatic precipitation	Cell type	Test atmosphere	Literature
Cultex CG	No	LK004 HFBE-21 CHO-K1 A549 BEAS-2B	Cigarette smoke Diesel exhaust Ozone and nitrogene dioxide Phosgene Volatile organic compounds Pharmaceuticals Trichloramine Fly ash Particles	Aufderheide and Mohr 1999 [12] Ritter et al. 2001 [46] Knebel et al. 2002 [47] Diabaté et al. 2008 [48] Pariselli et al. 2009 [49] Deschl et al. 2011 [50] Schmalz et al. 2011 [51] Wijte et al. 2011 [52] Nara et al. [53] Elihn et al. 2012 [54]

Cultex RFS	Yes	A549 16HBE14o-	Cigarette smoke	Aufderheide et al. 2011 [17]

ALICE	No	A549	Carbon black nanoparticles Zinc oxide nanoparticles Gold nanoparticles	Lenz et al. 2009 [14]

NACIVT	Yes	BEAS-2B Porcine lung macrophages	Secondary organic aerosols Polystyrene particles	Gaschen et al. 2010 [55] Savi et al. 2008 [16]

Vitrocell	No	A549	Laser printer emissions Volatile organic compounds Carbon nanotubes	Tang et al. 2012 [56] Frohlich et al. 2012 [57] Anderson et al. 2010 [58] Gminski et al. 2011 [59]

BAT	No	NCI-H292	Cigarette smoke	Phillips et al. 2005 [35]

EAVES	Yes	A549	Polystyrene particles Diesel exhaust Coarse ambient particles	de Bruijne et al. 2009 [15] Volckens et al. 2009 [60]
